# Quantitative Scrutinization of Aflatoxins in Different Spices from Pakistan

**DOI:** 10.1155/2016/4907425

**Published:** 2016-10-03

**Authors:** Narjis Naz, Aiza Kashif, Kinza Kanwal, Abdul Muqeet Khan, Mateen Abbas

**Affiliations:** ^1^Department of Chemistry, Lahore College for Women University, Lahore, Pakistan; ^2^Department of Pharmacology and Toxicology, University of Veterinary and Animal Sciences, Lahore, Pakistan; ^3^Department of Toxicology, Quality Operations Laboratory, University of Veterinary and Animal Sciences, Lahore, Pakistan

## Abstract

The current research work aimed to access the contamination level of aflatoxins B1, B2, G1, and G2 in the household spices that are widely consumed in huge amounts. 200 different spice samples, 100 packed and 100 unpacked, were analyzed for the aflatoxins profile by HPLC with an incidence of 61.5% contamination out of which 53.66% samples exceed the EU limit. The results disclosed that the unpacked samples are more contaminated as compared to the packed samples except for white cumin seeds. Among packed and unpacked samples of spices, the maximum value of aflatoxins was detected in fennel, that is, 27.93 *μ*g/kg and 67.04 *μ*g/kg, respectively. The lowest concentration of aflatoxin was detected in cinnamon in packed form (0.79 *μ*g/kg) and in the unpacked samples of white cumin seeds which is 1.75 *μ*g/kg. Caraway seeds and coriander in its unpacked form showed positive results whereas black pepper (packed and unpacked) was found free from aflatoxins. This is the first report on the occurrence of aflatoxins in packed and unpacked samples of spices from Pakistan. To ensure safe consumption of spices, there should be constant monitoring of aflatoxin and more studies need to be executed with the intention of preventing mycotoxin accretion in this commodity.

## 1. Introduction

Mycotoxins are toxic secondary metabolites which are produced by fungal spores [1, 2]. They can be entered into the living body through different natural routes; that is, they may be ingested, absorbed through skin, or inhaled via the nose [3, 4]. Due to their versatile nutritional requirements, they easily spread on different useful commodities under favorable conditions of humidity and temperature as noxious waste [5, 6]. Aflatoxins are the most extensively reviewed mycotoxin which is considered as the treacherous contaminants as far as human health is concerned [7–9]. The detrimental effects of aflatoxins include genotoxicity, teratogenicity, and immunosuppressive activity [10–12]. Aflatoxins are also considered as natural toxin of a variety of agricultural products [13–15]. The most prominent contamination has been faced in corn, peanuts, pistachio nuts, chestnuts, cottonseed, wine-grapes, spices, and other grain crops [16–18]. The pyramid of toxicity, carcinogenicity, and mutagenicity of different aflatoxins is in the order AF B1 > AF G1 > AF B2 > AF G2 [19, 20].

The important group of agricultural vendible is the spices which are used all over the world for gastronomic purposes, that is, as an ingredient to flavor different types of prepared food items or drinks, as ingredients of medicine, perfumes, and incense, and as a condiment. They are good not only for our taste but also for our health [21]. Their contamination with fungus is one of the major problems that can affect the human well-being and also degrade the quality and taste of the spices [22]. Owing to the substantial health jeopardies linked with the manifestation of aflatoxins in spices, the analysis of mycotoxin especially aflatoxins in spices is the perplexing task for the researchers nowadays due to the significant health risks associated with them.

The objective of this study was to appraise the prevalence level of aflatoxin contamination in common spices and to highlight their risk assessment. Two hundred packed and unpacked spices that are widely consumed in Pakistan were analyzed by HPLC using a C18 column and Fluorescence Detector after immunoaffinity column clean-up to establish a data collection on the occurrence of these toxins in habitually used spices. The literature reveals that high-performance liquid chromatography is the most frequently and widely used method for the mycotoxins analysis. It is quite sensitive and has a reasonably low level of detection of the number of toxins that have natural fluorescence like AFs, OTA, and so forth [23]. In spite of different exploration on mycotoxins in agricultural products from many countries, almost no information is accessible on aflatoxin contamination in different spices from Pakistan. The outcomes will give some significant references to highlighting the danger appraisal and investigating the quality of spices regarding aflatoxin contamination.

## 2. Experimental

### 2.1. Samples

A total of two hundred spices samples (*n* = 200) were purchased randomly from different supermarkets and bazaars located in Pakistan, from September to December 2014. The samples corresponded to 100 packed and 100 unpacked spices including cinnamon, caraway seeds, coriander, fennel, turmeric, black pepper, white pepper, black cumin seeds, white cumin seeds, and carom seeds. The samples were ground and 40 grams of each sample was stored in plastic bags, in the darkest conditions, at low relative humidity and at −4°C before the analysis for mycotoxins. Spice sampling was done in accordance with sampling provision described in European regulation number 401/2006. All samples were extracted and analyzed in triplicate.

### 2.2. Chemicals and Reagents

HPLC grade acetonitrile, methanol, TFA, and glacial acetic acid were purchased from Merck (Germany). Double distilled water was used for the preparation of solutions. Analytical standards of aflatoxins (AFB1, AFB2, AFG1, and AFG2) were purchased from Sigma-Aldrich (98% purity). Working standard solutions of aflatoxins having the concentration of 25 *μ*g/kg of AFB1 and AFG1 and 7.5 *μ*g/kg of AFB2 and AFG2 were prepared in the mixture of methanol and acetonitrile. HPLC eluents were digested for 5 min and filtered through mixed cellulose ester 0.45 mm filters (Advantec MFS, Pleasanton, CA, USA) before analysis. The laboratory glassware used was kept at 10% (v/v) nitric acid (Merck, Germany) overnight and rinsed several times with ultra-pure water before use.

### 2.3. Analysis of Aflatoxins in Spices

*Cleanup of Aflatoxins*. In the first part of this research, regarding the validation of the extraction method, two samples for each matrix, confirmed to be aflatoxin-free, were used as follows: one aliquot of the sample was analyzed as such, while the other aliquots were spiked with a known concentration of mycotoxin standard.

25 grams of the homogenized spice sample, added to 1 g of NaCl, was extracted in 100 mL of acetonitrile : water solution (84 : 16 v/v). Suspensions were shaken for 1 hour at using Ultra-Turrax Homogenizer (Polytron, Switzerland). Homogenized solutions were filtered through Whatman number 1 filter paper. Filtrate (9 mL) was diluted in acetic acid (24 mL) and applied to an Aflatest WB immunoaffinity column (Vicam, USA) at a flow rate of 1 mL/min. The column was then washed with distilled water (30 mL) and aflatoxins were eluted with methanol (2 mL) in to amber vials. After evaporation to dryness at 40°C under a stream of N_2_, the dry residue was redissolved in 20 *μ*L n-hexane and 50 *μ*L of TFA (trifluoroacetic acid) and shaken well. Then, 1.95 *μ*L of acetonitrile : water solution (10 : 90 v/v) was added in each bottle and placed for 5 min for the separation of layers. After 5 min, the mixture was poured in the HPLC sample vials with the help of Millex PTFE 0.45 mm (Millipore, USA) for aflatoxin analysis.

### 2.4. HPLC Parameters

An Agilent 1100 series HPLC system (Agilent Technologies, USA) was used with a fluorescence detection set at 360 nm excitation and 440 nm emission for aflatoxins G1 and G2 and 425 nm emission for aflatoxins B1 and B2. The mobile phase used for aflatoxin analysis was water : methanol : acetonitrile (300 mL : 100 mL : 100 mL v/v/v). The flow rate was 1 mL/min and column temperature was 40°C. A mixture of aflatoxin standards was used for construction of a five-point calibration curve of peak areas versus concentration (*μ*g/L). The injection volume was 20 *μ*L for both standard solution and sample extracts.

### 2.5. Method Validation

Validation of method was enhanced by reviewing the limit of detection (LOD) and limit of quantification (LOQ), based on the IUPAC definition. To validate the extraction method and chromatographic performances, repeatability of recovery (RSD) was calculated. The RSD, LOD, and LOQ were determined for each spice sample separately. Samples were spiked with three concentrations of standard solutions of aflatoxins AFB1, AFB2, AFG1, and AFG2 at 2, 6, and 10 *μ*g/kg of standard solutions. RSD and mean% were calculated, by analyzing spiked spice samples at 2, 6, and 10 *μ*g/kg. Each test was performed three times. LOD was defined as three times the electronic baseline noise and LOQ as ten times the level of the baseline noise. The baseline noise was obtained with a blank sample for each matrix processed following the tested procedures. Linearity was assessed using 8-point calibration curves prepared by injecting the calibration solutions and was defined using correlation coefficient (*R* ^2^) and slope. The following calibration solutions were prepared: AFB1 and AFG1 (0.2–35 ppb) and AFB2 and AFG2 (0.2–8 ppb). The linearity studies were conducted in triplicate. The precision was evaluated based on RSD.

## 3. Statistical Analysis

All the measurements of the packed and unpacked samples of spices were replicated three times and the data was statistically analyzed. Regression analyses were applied to find out the coefficient of determination (*R* ^2^). A Student's paired* t*-test was applied to analyze the differences between the AF level in packed and unpacked samples of spices using software IBM SPSS Statistics version 20. The calibration curves used for quantification were calculated by least-squares method. Samples with a concentration of AFs higher than LOD were considered positive, while the samples with concentrations lower than LOD were considered negative.

## 4. Results and Discussion

Culinary herbs are very sensitive to contamination with molds producing aflatoxin, so there is a need for detection and quantification of aflatoxins in spices. Aflatoxins continue to stance a health apprehension in human by exposure to these contaminated spices [24–26].

The analytical methods were validated considering linearity, standard deviation (SD), repeatability of recovery (RSD), limit of detection (LOD), and limit of quantification (LOQ). Data by performing analytical methods are summarized in Table 1.

LOD and LOQ obtained for white pepper were higher than the other spices tested and lie in the range of 0.14–0.47 *μ*g/kg and 0.30–2.35 *μ*g/kg, respectively. The results were explained to be due to the matrix compounds which interfere with the analytical signals increasing baseline noise. Recovery values for total AFs were above of 80% for all spices tested.

During this research, a total of 200 samples of spice including 100 packed and 100 unpacked samples were analyzed to evaluate the contamination level of aflatoxin in culinary herbs. 59% packed spices showed contamination with AFs out of which 34% samples displayed their toxicity level above the EU limit which is 10 *μ*g/kg for total AFs. The samples of black pepper, caraway seeds, and coriander showed negative results, whereas the incidence of aflatoxin was detected in 64% unpacked samples except for black pepper and 46% exceed the suggested limit.

The quantitative analysis of aflatoxin in spices showed that, in the packed samples, fennel contained the highest concentration of total aflatoxin, that is, 28.0 *μ*g/kg, followed by black cumin seeds (6.26 *μ*g/kg). The lowest concentration of 0.8 *μ*g/kg of total AFs was estimated in the sample of cinnamon. In the one hundred packed spice samples, seventeen samples showed AFB1 contamination (17%) and forty-two contained AFG1 (42%) whereas thirty-six unpacked spice samples were contaminated with AFB1 (36%) and eighteen contained AFG1 (18%) contamination, eighteen samples (18%) were contaminated with AFB2, and 10% were contaminated with AFG2.

The highest concentration in the unpacked samples of spices was detected in white pepper which was 89.50 *μ*g/kg of total aflatoxin while white cumin seeds exhibited the lowest concentration of 1.75 *μ*g/kg of aflatoxin B1. Eighteen unpacked samples (9%) and twenty-nine packed samples (14.5%) had a concentration below the suggested limit, whereas the remaining packed and unpacked samples had aflatoxin concentration within the limit recommended by the EU. The values of AFs are displayed in Tables 2 and 3.

**Figure 1:**
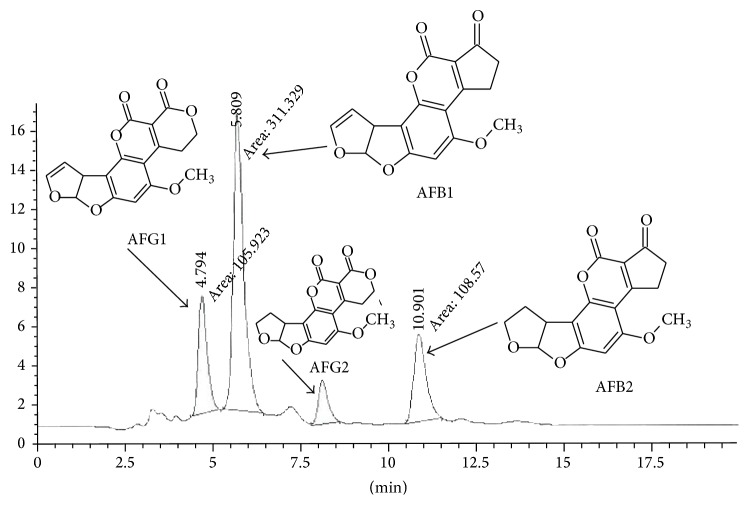
HPLC chromatogram of aflatoxin standards.

**Figure 2:**
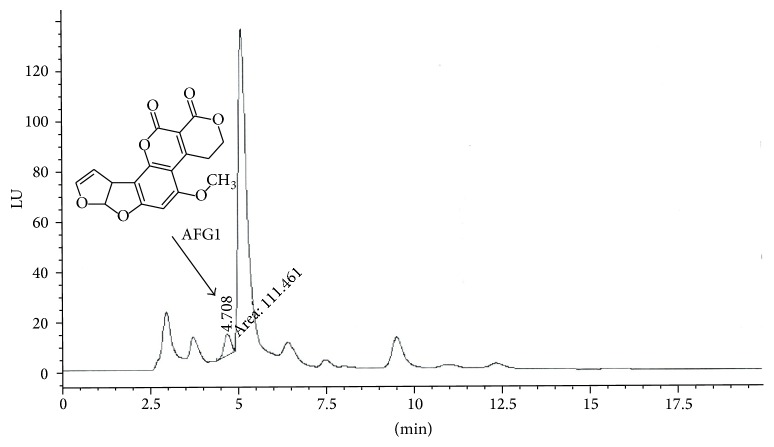
HPLC chromatogram of unpacked fennel sample.

The HPLC chromatograms were obtained when the standards and the samples were analyzed through the system and shown in Figures 1 and 2. Mycotoxins were identified by using retention time and compared with reference standards while the quantification was done by taking the mean values of peak areas.

**Figure 3:**
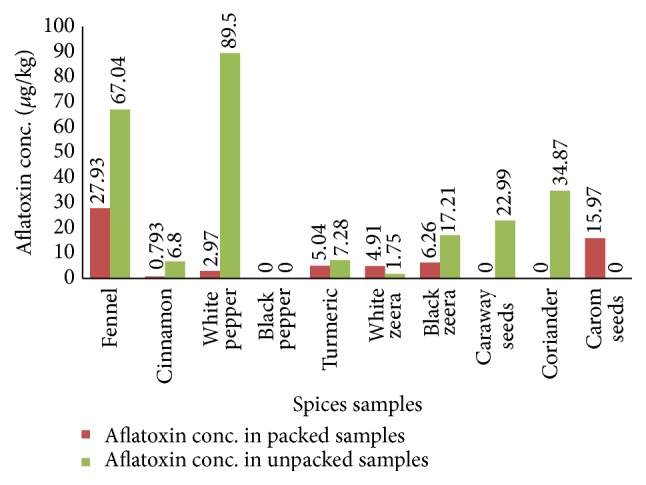
Comparison of aflatoxin conc. in packed and unpacked spice samples.

The results showed that unpacked spices were more contaminated than packed spices; however, the packed samples of white cumin seeds displayed opposite results. This high aflatoxin level might be due to the environmental factors during the drying and storing processes which may promote the growth of aflatoxins as compared to unpacked sample. The experimental data showed that the aflatoxin amount in the unpacked sample of white pepper (89.50 *μ*g/kg) was much greater than the packed form (2.96 *μ*g/kg), whereas the unpacked samples of fennel and black cumin seeds contained more aflatoxin concentration values 67.04 *μ*g/kg and 17.21 *μ*g/kg, respectively, than those of the packed sample of fennel and black cumin seeds of 27.93 *μ*g/kg and 6.26 *μ*g/kg correspondingly. The spices like caraway seeds (Kalonji) and coriander in their unpacked form showed aflatoxin contamination but did not give any mycotoxin detection in their packed form. Out of all these samples, some have aflatoxin concentration within the strict limitations set by the European Union which is 10 *μ*g/kg for total aflatoxins (European Mycotoxin Awareness Network (EMAN), WHO Food Additives Series 40, JECFA monograph on Aflatoxins) while most of the spices exceed the level set by the EU, which showed that they are highly contaminated with aflatoxins and are precarious for the health of people who consume them as a part of their food chain.  Figure 3 showed the comparison of the aflatoxin concentration in the packed and unpacked samples of different spices collected from different areas of Pakistan.

This study provides the first description of aflatoxins contamination of spices marketed in Pakistan, where the regulations have less impact on mycotoxins control as there is no strict check on concerns over food protection. The present research work provides useful information about the risk of mycotoxins hazards and creates the awareness among the consumers, researchers, and farmers to improve the processing methods including harvesting, drying, transportation, and storage conditions.

## 5. Conclusion

The current analysis is a paramount inclusive appraisal to reconnoiter the manifestation of aflatoxin contamination in the packed and unpacked samples of spices from Pakistan. It emerged that the contamination of spices with aflatoxins was detected to be higher than that recommended by the EU and this is obnoxious for the developing country to vie in the contemporary uncluttered arcade place. For consumer safety, the regulatory authorities should take into account this issue of contamination and quality control and quality assurance strategies should be implemented.

## Figures and Tables

**Table 1:** Recoveries of aflatoxin from spiked spice samples.

Samples	Spike level (*µ*g/kg)	2				6				10			
		B1	B2	G1	G2	B1	B2	G1	G2	B1	B2	G1	G2
Fennel	Mean%	91.7	84.3	77.5	80.7	88.9	70.5	91.9	92.9	96.2	95.7	96.3	95.8
	RSD%	2.58	2.08	1.71	2.93	1.70	2.02	1.17	1.83	1.24	2.13	2.36	1.07

Cinnamon	Mean%	87.3	82.0	93.3	91.2	90.3	96.1	97.3	90.7	95.5	96.6	97.9	97.0
	RSD%	1.44	2.79	2.53	1.68	0.74	2.18	2.62	2.12	1.05	0.47	1.02	1.77

White pepper	Mean%	92.3	82.5	79.5	90.9	94.5	95.5	90.8	89.5	97.6	97.9	94.2	91.5
	RSD%	2.78	1.82	1.89	2.84	2.31	1.26	2.02	2.82	1.23	1.02	1.68	2.56

Black pepper	Mean%	88.3	85.3	90.0	92.2	97.1	97.8	92.1	97.0	98.3	98.0	94.6	97.1
	RSD%	2.29	2.89	2.55	2.73	1.17	1.45	1.98	2.73	1.70	1.12	1.31	1.29

Turmeric	Mean%	89.3	89.8	81.3	77.8	95.4	96.5	94.4	92.6	97.0	98.3	95.7	95.4
	RSD%	1.97	2.63	2.91	2.26	1.31	0.69	1.43	1.80	1.20	1.09	1.15	1.13

White cumin seeds	Mean%	96.5	80.3	88.8	80.7	94.2	92.8	90.9	93.9	97.4	94.5	94.7	97.8
	RSD%	5.65	2.81	2.13	2.18	1.57	1.94	2.39	2.67	1.19	1.06	1.76	1.42

Black cumin seeds	Mean%	93.5	87.0	84.7	95.8	88.9	95.1	89.2	94.0	97.4	95.7	92.9	95.8
	RSD%	2.67	2.51	1.80	1.83	2.26	2.55	1.41	1.95	1.08	1.39	1.32	1.37

Caraway seeds	Mean%	91.5	89.3	82.0	92.3	95.6	97.6	96.1	94.2	96.7	97.8	96.4	96.1
	RSD%	2.89	1.97	2.79	2.44	1.58	1.42	2.09	1.68	1.79	2.13	1.16	2.15

Coriander	Mean%	94.3	89.8	81.3	87.8	95.4	90.9	89.4	90.9	97.0	93.6	94.7	95.4
	RSD%	1.86	2.63	2.91	2.00	1.31	1.73	1.51	1.74	1.20	1.82	0.75	1.13

Carom seeds	Mean%	92.2	85.3	90.0	92.2	97.1	97.8	92.1	97.0	98.3	98.0	94.6	97.1
	RSD%	2.73	2.89	2.55	2.73	1.17	1.45	1.98	2.53	1.70	1.12	1.31	1.29

**Table 2:** Incidence of aflatoxins in packed spice samples.

Sample	Number of packed samples (*n*)	Number of AF- contaminated samples	Mean of AFB1	Mean of AFB2	Mean of AFG1	Mean of AFG2	Total AFs
			Mean (*µ*g/kg) ± SD	Mean (*µ*g/kg) ± SD	Mean (*µ*g/kg) ± SD	Mean (*µ*g/kg) ± SD	Mean (*µ*g/kg) ± SD
Fennel	10	10	—	—	27.93 ± 0.37	—	27.93 ± 0.37
Cinnamon	10	06			0.79 ± 0.12		0.79 ± 0.12
White pepper	10	10	—	—	2.96 ± 0.20		2.96 ± 0.20
Black pepper	10	00	ND	ND	ND	ND	ND
Turmeric	10	07	—	—	5.04 ± 0.16		5.04 ± 0.16
White cumin seeds	10	09	—	—	4.91 ± 0.23		4.91 ± 0.23
Black cumin seeds	10	07	6.26 ± 0.06				6.26 ± 0.06
Caraway seeds	10	00	ND	ND	ND	ND	ND
Carom seeds	10	00	ND	ND	ND	ND	ND
Coriander	10	00	ND	ND	ND	ND	ND

**Table 3:** Incidence of aflatoxins in unpacked spice samples.

Sample	Number of unpacked samples (*n*)	Number of AF- contaminated samples	Mean of AFB1	Mean of AFB2	Mean of AFG1	Mean of AFG2	Total AFs
			Mean (*µ*g/kg) ± SD	Mean (*µ*g/kg) ± SD	Mean (*µ*g/kg) ± SD	Mean (*µ*g/kg) ± SD	Mean (*µ*g/kg) ± SD
Fennel	10	09	—	—	67.04 ± 1.74	—	67.04 ± 1.74
Cinnamon	10	06	6.80 ± 0.33				6.80 ± 0.33
White pepper	10	10				89.50 ± 2.62	89.50 ± 2.62
Black pepper	10	00	ND	ND	ND	ND	ND
Turmeric	10	07	7.28 ± 0.35				7.28 ± 0.35
White cumin seeds	10	05	1.75 ± 0.17				1.75 ± 0.17
Black cumin seeds	10	09	3.69 ± 0.22		13.52 ± 0.72		17.21 ± 0.94
Caraway seeds	10	09		22.99 ± 1.00			22.99 ± 1.00
Carom seeds	10	10	15.97 ± 0.28				15.97 ± 0.28
Coriander	10	09	4.05 ± 0.34	30.82 ± 2.54			34.86 ± 2.88
